# Supplementary material for: Towards an understanding of spiral patterning in the Sargassum muticum shoot apex
Source: Sci Rep. 2017 Oct 24;7:13887. doi: 10.1038/s41598-017-13767-5 (PMC5654765; doi:10.1038/s41598-017-13767-5)
Supplement: Supplementary file 1 — Supplementary Figures [file 41598_2017_13767_MOESM1_ESM.pdf]

Towards an understanding of spiral patterning in the *Sargassum muticum* shoot apex

Authors: Marina Linardić and Siobhan A. Braybrook

Supplementary Information

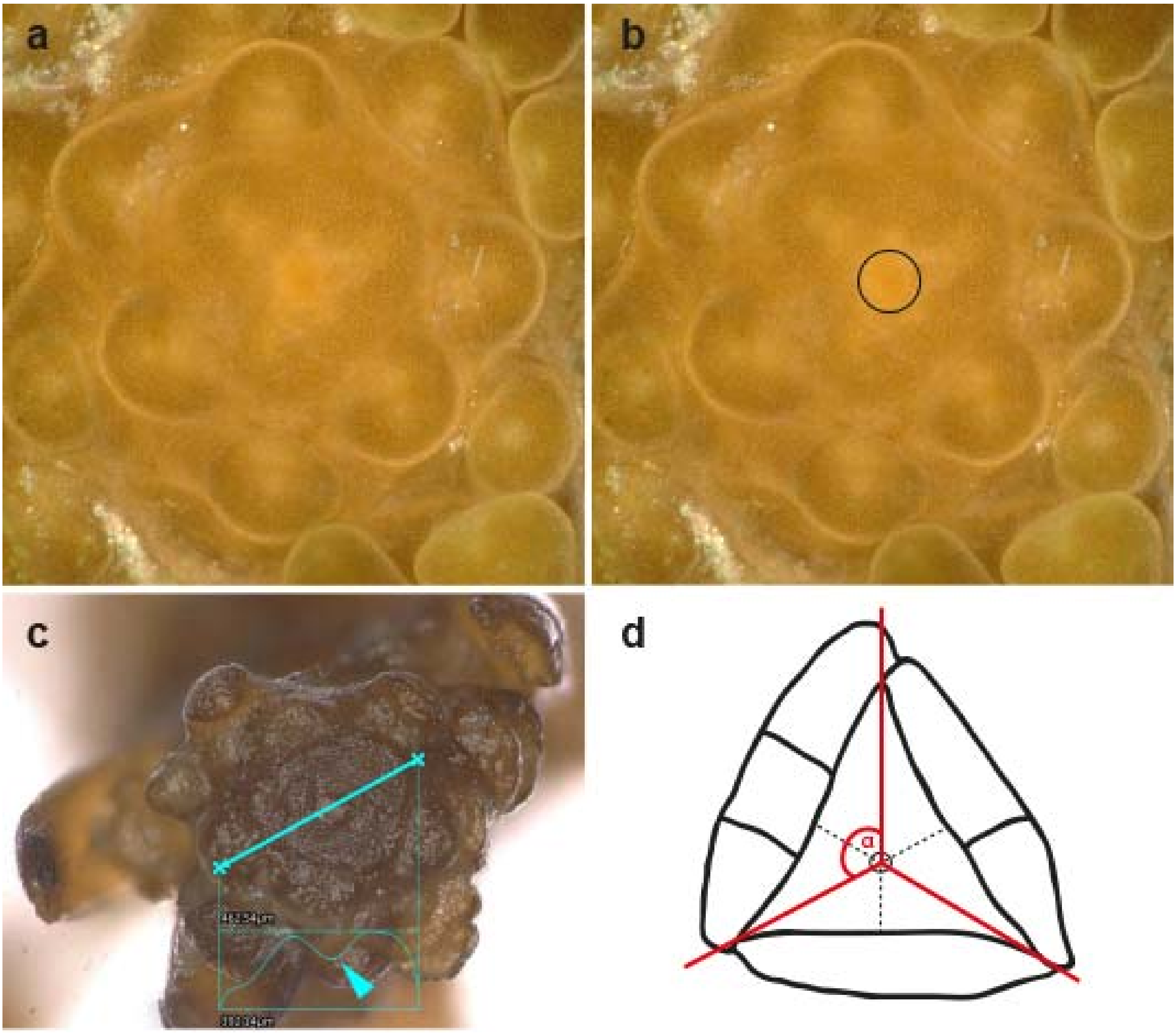

Supplementary Figure 1. **Analysis processes for meristem zones and apical cell division angles.** (a) Raw image of a meristem showing lighter colour in the middle (area where the apical cell is positioned); (b) same image with a black circle around the approximated apical pit area. (c) 3D image of a Sargassum apex with a topological line drawn through the middle; blue arrow points and the apical pit. (d) Scheme of a transverse section of an apical cell and daughter cells showing the method of determining the division process: apical cell is seen as an equilateral triangle; dotted lines correspond to the altitude for each triangle side whose crossing point determines the centre. The division angle is measured by drawing 2 lines from the centre through the two interfaces which the daughter cell shares with its neighbouring cells.

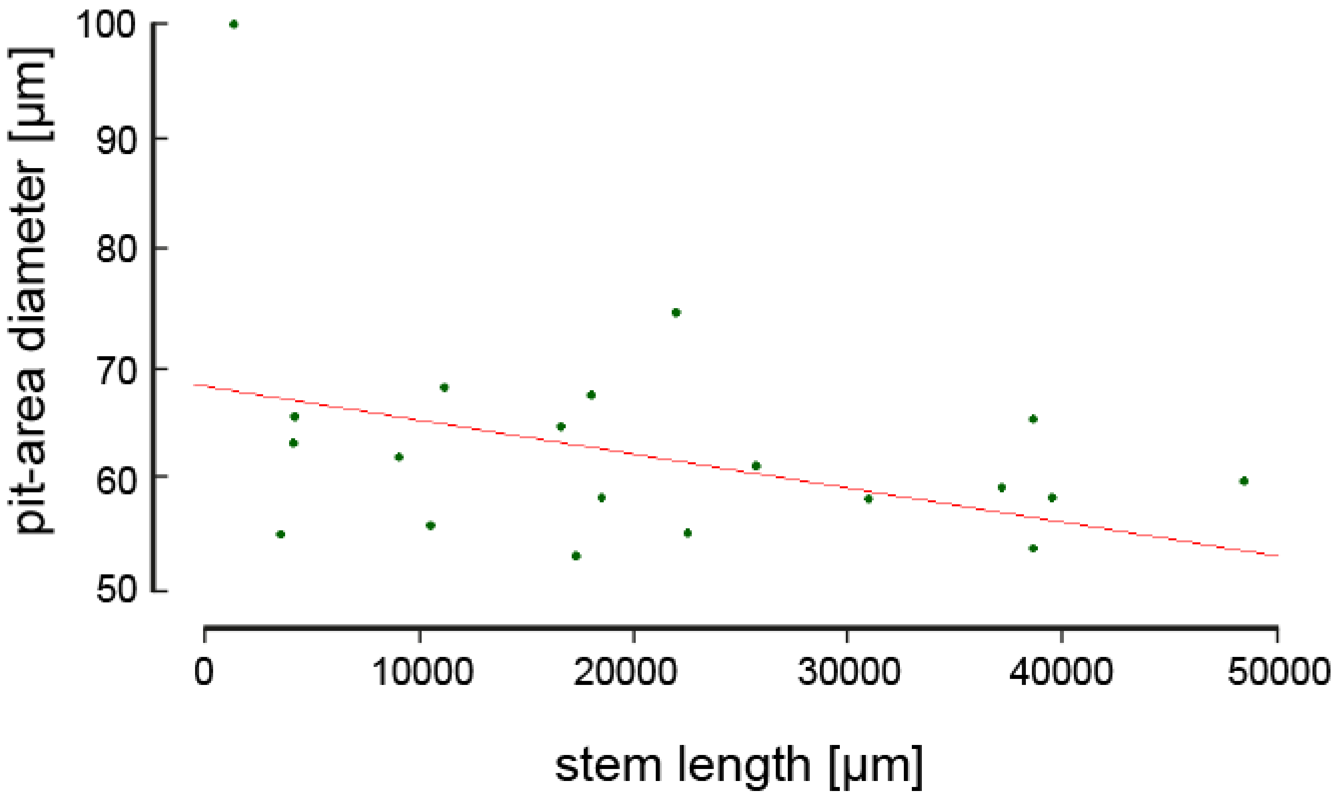

Supplementary Figure 2. **The age of the stipe and the meristem area.** Scatter plot showing a lack of correlation between the length (proxy for age) of an individual stipe with the diameter of its pit-area (proxy for meristem size; apical cell and the promeristem cells around it) (n=22, p-value=0.07, r=-0.39; two-sample t-test).

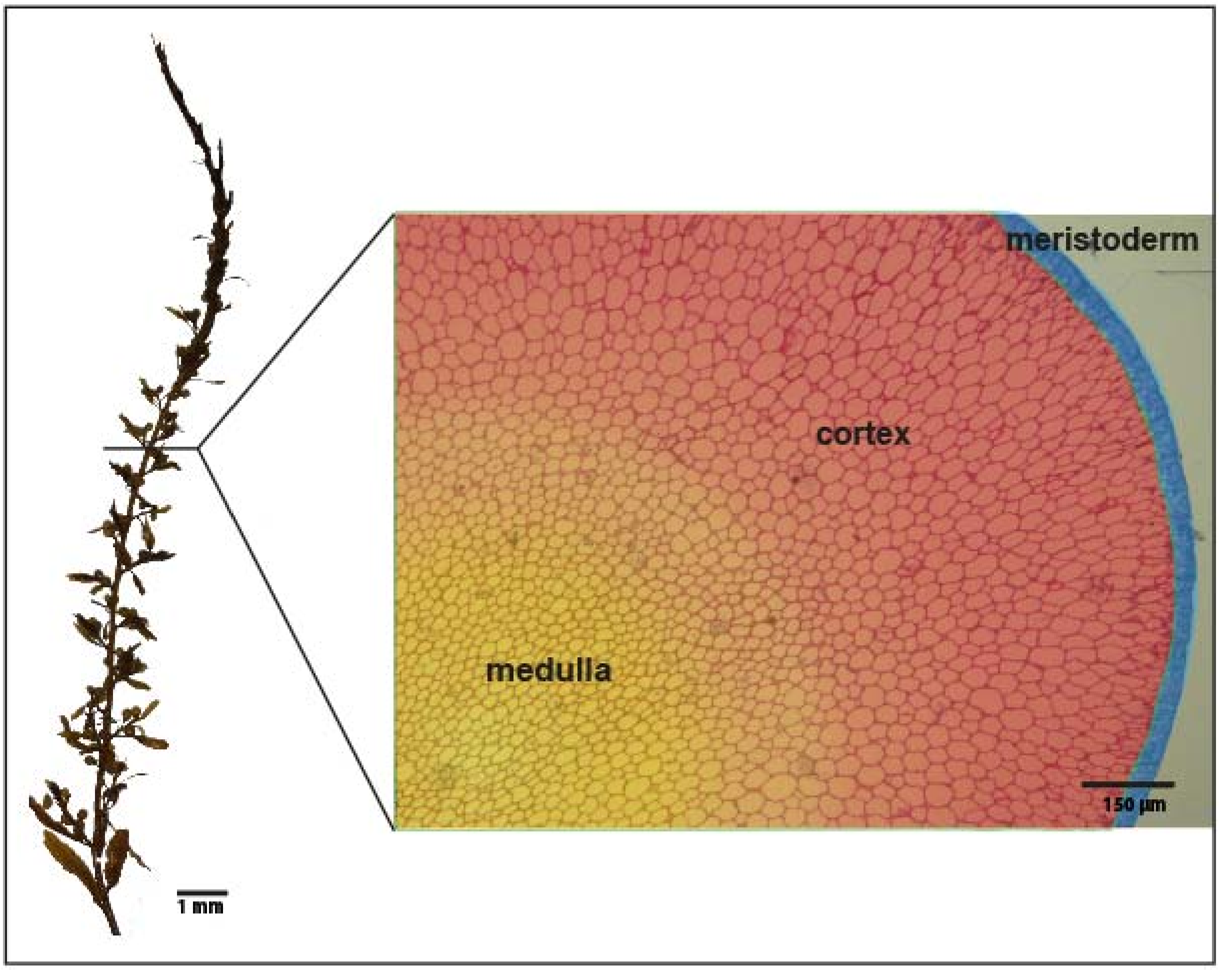

Supplementary Figure 3. **Types of tissues found in *S.muticum* as illustrated on a stipe section.** Outer layer (meristoderm), middle layer (cortex) and inner layer (medulla). Scale bar 150 µm, 1 mm (whole algal body).

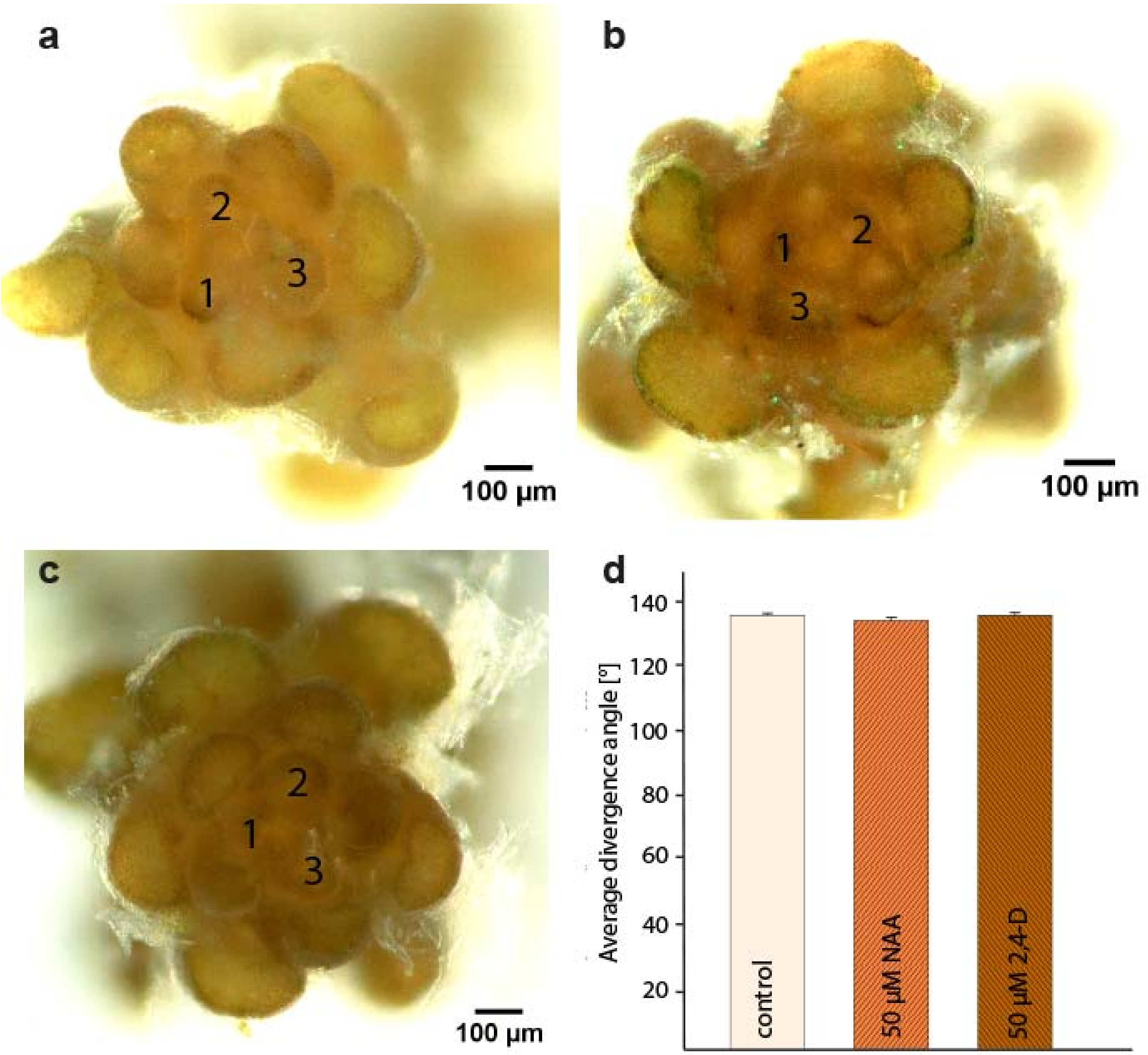

Supplementary Figure 4. **The effect of exogenous auxin on *S.muticum* phyllotaxis.** When compared to control treatments (a, 50µM DMSO), neither IAA (b) or 2,4-D (c) auxins influenced phyllotactic patterning when supplied to the apices at 50µM in artificial sea water. Divergence angle was quantified for n=20 apices per treatment (d).

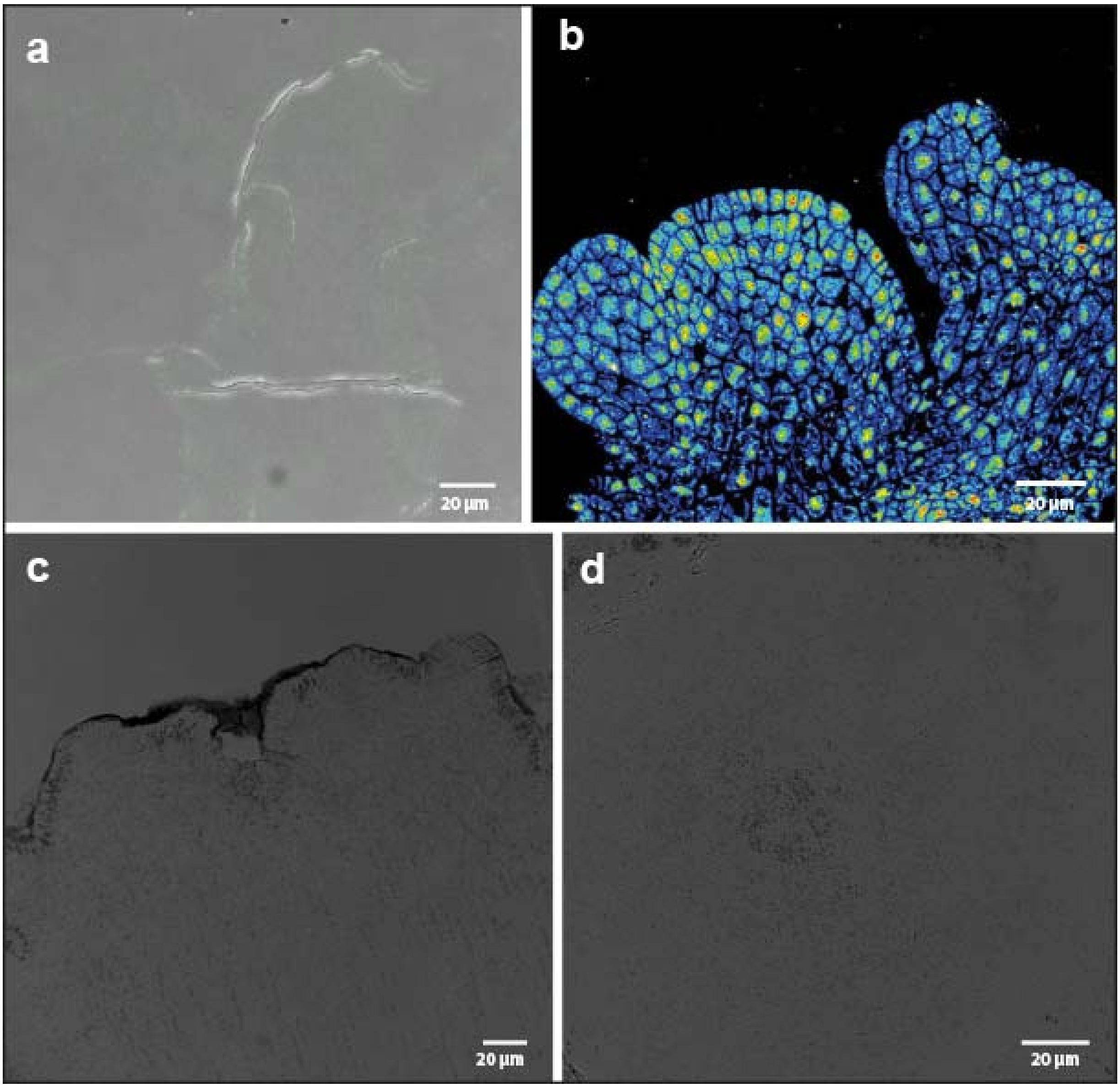

Supplementary Figure 5. **Control confocal images for the auxin and alginate immunolocalisations.** *Arabidopsis thaliana* longitudinal section with no primary antibody control (a) and anti-IAA (b). (c) No primary antibody controls of S. muticum apex sections for alginate immunolocalisation (c) longitudinal section and (d) transverse. All controls merged with a bright-field image for visualisation. Scale bar 20 µm.
